# Reports on Cities, Towns, and Districts

**Published:** 1857-01

**Authors:** George Rigden

**Affiliations:** Surgeon to the Canterbury Dispensary


### SANITARY AND SOCIAL SCIENCE.

### REPORTS ON CITIES, TOWNS, & DISTRICTS.

### REPORT ON THE SANITARY STATE OF THE CITY AND BOROUGH OF CANTERBURY, PARTICULARLY DURING THE YEARS 1854 AND 1855.

By GEORGE RIGDEN, M.R.C.S., Surgeon to the Canterbury Dispensary

(*Concluded from* p. 175.)

TABLE I. is intended to represent the different parishes, and portions of parishes, comprised within the boundaries of the city of Canterbury; the first fourteen on the list are included in the poor-law district of Canterbury; the 15th, 16th, 17th, and 18th are portions of parishes belonging to the poor-law district of Bridge; the remaining parishes, and parts of parishes, are united to the poor-law district of Blean.

TABLE I. *Parishes included in the City of Canterbury.*

| | Inhabited houses in 1851. | Population in 1851. | Births in 1854 and 1855. | Deaths in 1854 and 1855. |
|---|---|---|---|---|
| CANTERBURY POOR-LAW DISTRICT. | | | | |
| 1. St. Mary Northgate ...... | 496 | 3,091 | 182 | 222 |
| 2. St. Mildred ............... | 452 | 2,003 | 119 | 90 |
| 3. St. Paul ................ | 341 | 1,729 | 96 | 121* |
| 4. St. Alphage ............... | 238 | 1,098 | 70 | 55 |
| 5. St. Peter ................. | 255 | 1,198 | 68 | 52 |
| 6. St. Andrew .............. | 92 | 538 | 17 | 12 |
| 7. St. Mary Bredman ........ | 71 | 391 | 19 | 9 |
| 8. St. Mary Bredin .......... | 175 | 1,007 | 47 | 82 |
| 9. St. George .............. | 252 | 1,186 | 50 | 44 |
| 10. All Saints................ | 72 | 412 | 16 | 20 |
| 11. St. Margaret ............ | 108 | 620 | 16 | 27 |
| 12. St. Mary Magdalen......... | 81 | 413 | 15 | 9 |
| 13. St. Martin ............. | 45 | 189 | 15 | 11 |
| 14. Holy Cross, Westgate Within | 50 | 225 | 10 | 6 |
| BRIDGE POOR-LAW DISTRICT. | | | | |
| 15. HolyCross,WestgateWithout | 159 | 853 | 44 | 50 |
| 16. Part of Thanington ...... | — | 158 | 7 | 3 |
| 17. „ Nackington ...... | — | 20 | — | — |
| 18. „ Patrixbourne ...... | — | 13 | — | — |
| BLEAN POOR LAW DISTRICT. | | | | |
| 19. St. Dunstan.............. | — | 1,246 | 75 | 60 |
| 20. St. Gregory .............. | 270 | 1,278 | 91 | 69 |
| 21. Christ Church ............ | 38 | 243 | 4 | 3 |
| 22. Staplegate (extraparochial) | 67 | 261 | 11 | 7 |
| 23. Archbishop's Palace (ditto) | 32 | 183 | 2 | — |
| 24. Part of St. Stephen's ...... | — | 43 | 1 | 12 |
| Totals ............... | — | 18,398 | 975 | 964 |

* During the year 1854, nine males and nine females, and during the year 1855, twenty-two males and ten females, died in the Kent and Canterbury Hospital, situate in the parish of St. Paul's, who had been brought from parishes unconnected with the city of Canterbury.

To each parish, with the exception of those of which only a portion is included in the city, and of which no separate returns could be procured, there is shown the number of inhabited houses, and population according to the last census; together with the births and deaths in the two years 1854 and 1855. In the parish of St. Mary's, Northgate, are situated the Military Barracks, containing, when the last census was taken, but 383 soldiers; whereas, during the last year, there have often been as many as 2,000 stationed there. During the year 1854, twenty-eight deaths—and in the year 1855, thirty-two deaths occurred in barracks; this has necessarily increased the number of deaths, and, in some degree, of births in that parish, in proportion to its recorded population. In the parish of St. Paul's is situated the Kent and Canterbury Hospital, having within its walls upon an average eighty-five patients. During the year 1854, twenty-nine deaths were registered as occurring in that institution, of whom eleven only belonged to the city parishes; in the year 1855 there were fifty deaths, of which eighteen only belonged to the city parishes. The deaths of those belonging to the city have been placed against the parish to which they properly belonged; but this will leave a surplus of fifty deaths occurring in the parish of St. Paul's, the subjects of which had been brought thither, in a state of disease or injury, from the neighbouring districts. It is not improbable that where the parishes are dovetailed into each other, the persons resident upon the margins are occasionally deceived as to which parish they are actually resident in; and, again, where there is no district parish church, as in Staplegate and the precincts of the archbishop's palace, it is not unusual for the more humble classes to consider themselves as belonging to St. Alphage, or to the nearest parish to the church of which they commonly attend for worship. These circumstances may have occasioned an undue number of births or deaths to become registered in such parishes in proportion to their population; but such mistakes cannot have occurred at the margins of the city as its limits are more strictly defined. Again, although it is impossible that deaths can, it is not improbable that some births escape registration; but from the general activity of our local registrars, and from the limited and almost permanent nature of our population, there is no reason to suspect that this can occur frequently, or, at all events, more frequently than in other districts. It is, however, evident that the number of births in proportion to the population, even of 1851, is exceedingly small. It is difficult to account for this unless upon the supposition that many of the citizens either join the army or navy, or emigrate into other districts just previously to attaining mature age, and this receives corroboration from the fact that in comparison with other towns there is an excess of population at young and advanced ages, and a deficiency at adult and middle ages in Canterbury. (*Vide* population tables of Canterbury and other towns, *Association Medical Journal*, July 1855.)

Table II. is intended to represent, in each month of each year, the number of births and deaths; the highest, lowest, and mean temperature; the highest and lowest points noted by the barometer; the fall of rain, in inches and 100th parts of inches; and the number of days in which the wind has blown from each point of the compass.

The law allowing parents, guardians, or householders to register a death at any time previously to burial, and a birth, without payment, at any time within six weeks of its occurrence, it frequently happens that the month of registry has not been the month in which the birth or death occurred: pains have therefore been taken in the preparation of this table to prefix each to its proper month. The atmospheric observations have all been taken at 8½ A.M. daily, as nearly as possible in the centre of the city; the highest temperature from a thermometer, placed five feet from the ground against a wall having a west aspect; the lowest temperature from a register thermometer, showing the lowest point since the last observation, placed five feet from the ground against a small tree with a south aspect; the mean temperature from the daily observation of the two thermometers. The fall of rain is noted at the same locality. The direction of the wind is noted from the vanes on the Bell Harry tower of the cathedral. "Fog" is noted when the vanes cannot be seen from that cause; "differ" when the air is calm, and the vanes at the same altitude point in different directions; and in a few days in each year, from unavoidable circumstances, no note has been made. (See next page.)

*Diseases.* For the purposes of this report the registers of births and deaths have been examined monthly: with respect to the latter, the cause of death has been in each case verified or corrected from the certificates supplied by the medical practitioners in attendance. The nomenclature adopted has been exactly that used upon the medical certificate; and the classification that adopted by the Registrar-General.

In the two years, 1854 and 1855, the deaths resulted from the following causes. (See page 387.)

### Table II.

| 1854. | Births. | | Deaths. | | Temperature. | | | Barometer. | | Rain in inches and 100th parts of inc. | Direction of the Wind. | | | | | | | | | | | |
|---|---|---|---|---|---|---|---|---|---|---|---|---|---|---|---|---|---|---|---|---|---|---|
| | Males. | Females. | Males. | Females. | Highest. | Lowest. | Mean. | Highest. | Lowest. | | North. | East. | West. | South. | North-east. | North-west. | South-east. | South-west. | Fog. | Vanes differ. | Not noted. | |
| January ...... | 24 | 20 | 13 | 17 | 52 | 17 | 36 | 30·42 | 28·77 | 2·44 | 3 | 3 | 6 | 6 | 1 | 1 | 1 | 9 | 1 | — | — | days |
| February ...... | 20 | 21 | 22 | 13 | 47 | 27 | 35 | 30·50 | 29·77 | 1·48 | 2 | 1 | 6 | 1 | — | 9 | 1 | 7 | 1 | — | — | „ |
| March ........ | 28 | 19 | 20 | 12 | 52 | 23 | 38 | 30·59 | 29·78 | ·63 | 5 | 3 | 4 | 3 | 3 | — | 2 | 11 | — | — | — | „ |
| April .......... | 14 | 22 | 16 | 19 | 58 | 31 | 44 | 30·40 | 29·50 | ·65 | 8 | 7 | 2 | 2 | 5 | 1 | — | 5 | — | — | — | „ |
| May .......... | 31 | 27 | 15 | 10 | 57 | 35 | 46 | 30·10 | 29·11 | 2·81 | 6 | — | 4 | 3 | 2 | — | 1 | 15 | — | — | — | „ |
| June .......... | 15 | 15 | 24 | 14 | 69 | 41 | 54 | 29·98 | 29·54 | 1·20 | 3 | 1 | — | 3 | 5 | — | 1 | 9 | — | 1 | 7 | „ |
| July .......... | 23 | 13 | 17 | 18 | 70 | 38 | 56 | 30·12 | 29·66 | 2·13 | 1 | 1 | 4 | 3 | 3 | — | 3 | 7 | — | — | 9 | „ |
| August ........ | 23 | 18 | 38 | 38 | 68 | 42 | 60 | 30·34 | 29·58 | 3·44 | 8 | — | 1 | 3 | 3 | 3 | — | 13 | — | — | — | „ |
| September .... | 14 | 21 | 35 | 32 | 66 | 43 | 53 | 30·30 | 29·72 | ·92 | 3 | 4 | 3 | 4 | 4 | 3 | 3 | 6 | — | — | — | „ |
| October ...... | 18 | 16 | 24 | 21 | 56 | 33 | 44 | 30·33 | 29·06 | 5·49 | 1 | 2 | 4 | 5 | 2 | 5 | 1 | 10 | — | — | 1 | „ |
| November .... | 14 | 27 | 18 | 17 | 57 | 27 | 37 | 30·36 | 28·90 | 2·50 | 4 | 2 | 9 | — | 3 | 5 | 3 | 3 | 1 | — | — | „ |
| December .... | 28 | 22 | 19 | 11 | 52 | 28 | 37 | 30·32 | 29·25 | 2·02 | — | — | 15 | — | — | 6 | — | 10 | — | — | — | „ |
| | | | | | | | | | | | 44 | 24 | 58 | 33 | 31 | 33 | 16 | 105 | 3 | 1 | 17 | 365 |
| 1855. | | | | | | | | | | | | | | | | | | | | | | |
| January ...... | 23 | 22 | 24 | 34 | 48 | 17 | 33 | 30·41 | 29·43 | ·78 | 4 | 7 | 3 | 1 | 2 | 5 | 2 | 7 | — | — | — | „ |
| February ...... | 20 | 14 | 29 | 32 | 41 | 12 | 26 | 29·88 | 29·16 | 1·35 | 1 | 8 | 2 | — | 8 | 2 | 3 | 2 | 2 | — | — | „ |
| March ........ | 31 | 15 | 35 | 23 | 48 | 25 | 34 | 30·28 | 28·80 | 2·23 | 8 | 3 | 5 | 3 | 7 | — | 1 | 3 | 1 | — | — | „ |
| April .......... | 16 | 20 | 24 | 24 | 54 | 29 | 40 | 30·40 | 29·24 | ·25 | 6 | 1 | 6 | 2 | 8 | 3 | — | 3 | — | 1 | — | „ |
| May .......... | 8 | 22 | 17 | 19 | 66 | 32 | 44 | 29·94 | 29·42 | 2·00 | 8 | 1 | 6 | 5 | 5 | 2 | 1 | 3 | — | — | — | „ |
| June .......... | 21 | 17 | 15 | 17 | 69 | 42 | 53 | 30·10 | 29·50 | 1·77 | 1 | — | 5 | 1 | 1 | 2 | 1 | 1 | — | — | 18 | „ |
| July .......... | 22 | 19 | 13 | 14 | 68 | 49 | 59 | 30·12 | 29·46 | 2·80 | 1 | 1 | 9 | 6 | 3 | 2 | 1 | 7 | — | — | 1 | „ |
| August ........ | 20 | 13 | 14 | 14 | 68 | 49 | 58 | 30·14 | 29·58 | 1·67 | 2 | 1 | 7 | 7 | — | 3 | 1 | 10 | — | — | — | „ |
| September .... | 21 | 19 | 9 | 9 | 65 | 38 | 53 | 30·30 | 29·48 | ·83 | 8 | 2 | 1 | 3 | 8 | 1 | 1 | 6 | — | — | — | „ |
| October ...... | 25 | 13 | 29 | 16 | 58 | 40 | 49 | 30·07 | 29·18 | 4·17 | 1 | 1 | 3 | 6 | 1 | 1 | 3 | 15 | — | — | — | „ |
| November .... | 23 | 20 | 18 | 12 | 50 | 31 | 40 | 30·13 | 29·43 | 2·85 | 7 | 2 | 3 | 4 | 9 | 1 | 1 | 3 | — | — | — | „ |
| December .... | 36 | 22 | 18 | 22 | 47 | 18 | 33 | 30·17 | 29·20 | 2·32 | 1 | 3 | 10 | 9 | 1 | 3 | 4 | — | — | — | — | „ |
| Totals .... | 518 | 457 | 506 | 458 | | | | | | | 48 | 30 | 60 | 47 | 53 | 25 | 19 | 60 | 3 | 1 | 19 | 365 |

| | Males. | Females. | Totals. |
|---|---|---|---|
| 1. Zymotic or epidemic, endemic or contagious diseases | 128 | 117 | 245 |
| 2. Diseases of uncertain or variable seat - - | 7 | 17 | 24 |
| 3. Tubercular diseases - - - - - - | 68 | 51 | 119 |
| 4. Diseases of the nervous system - - - | 59 | 44 | 103 |
| 5. " organs of circulation - - | 13 | 22 | 35 |
| 6. " organs of respiration - - | 72 | 53 | 125 |
| 7. " organs of digestion - - - | 26 | 41 | 67 |
| 8. " urinary organs - - - | 14 | 7 | 21 |
| 9. " generative organs - - - | — | 3 | 3 |
| 10. " organs of locomotion - - | 3 | 1 | 4 |
| 11. Malformation - - - - - | — | 2 | 2 |
| 12. Premature birth - - - - - | 17 | 9 | 26 |
| 13. Atrophy - - - - - - | 10 | 9 | 19 |
| 14. Age - - - - - - - | 47 | 66 | 113 |
| 15. External causes - - - - - | 21 | 9 | 30 |
| No cause assigned - - - - - | 21 | 7 | 28 |

In Class 1 are included thirty-five deaths from small-pox, viz., twenty males and twenty-five females: these occurred in an epidemic extending from October 1854, to the end of the period here reported upon. With the exception of January and July, deaths are reported from this disease in each month of the year 1855. Eighteen of the deaths occurred in children under 10 years of age; two between 10 and 15 years; three between 15 and 20 years; eight between 20 and 25 years; three between 25 and 30 years; and one, a male, was between 40 and 45 years of age. Thirty-seven of the deaths in this class are reported as the result of scarlet-fever, viz., twenty-one males and sixteen females. This epidemic extended through the entire of the two years, but was most virulent during the months from July to December inclusive, in the year 1854. Thirty-four of the deaths occurred in children under 10 years of age; and one in each of the three next quinquennial periods of age. Two deaths only are reported from measles, both occurring in December 1855; they were in children under 5 years of age. Seven from hooping-cough, in children under 5 years of age; these occurred principally in November and December 1855. Two from croup and two from thrush. Twenty-one from diarrhœa, viz., nine males and twelve females; principally in September, October, and November, 1854; fourteen were in children under 10 years of age, one between 15 and 20 years, and the remainder were more than 50 years of age.

Sixty-one deaths, viz., thirty-two males and twenty-nine females, are reported as the result of cholera; one of these occurred in the Military Barracks, in January 1854; the remaining cases occurred in July, August, September, and October of the same year, with the exception of the period of between 55 and 60 years, at which age there are no deaths reported; the mortality seems very nearly equal at all other periods of life.

Ten occurred from influenza, viz., five males and five females, in the months of January, February, and March, 1855; with the exception of two children, the deaths from this disease occurred in persons more than 60 years of age. Four from purpura, in the months of April, August, and October, 1855; two of these were between the ages of 10 and 15 years of age, one between 40 and 45 years, and one between 45 and 50 years.

Fifty-one deaths resulted from fever, viz., twenty-seven males and twenty-four females, with the exception of January and June 1854, and November and December 1855, each month has produced deaths from this disease. No males between the ages of from 50 to 60, and no females between the ages of from 25 to 45, died from fever; with these exceptions no age has been exempt; but the greatest mortality was under 5 years of age and between the ages of 15 and 20 years.

Ten deaths from erysipelas, five males and five females, occurred in the months of March, October, November, and December, 1854, and April, May, October, and December, 1855; the ages of these were from 25 to 80 years.

Two males are reported to have died from syphilis, one under 5 years of age, and one between 40 and 45 years.

One female, under 5 years, in January 1854, is reported to have died from chicken-pock.

Class 2. Two males and six females died from dropsy; four males from abscess; one male and ten females from cancer; and one female from a sarcomatous tumour.

Class 3. Three males and five females died from scrofula; twelve males and six females from tabes mesenterica; fifty males and forty females from phthisis, or consumption; and three males from hydrocephalus. The deaths from the former two and the last disease were almost confined to children under 5 years of age; those from consumption occurred principally at the ages between 20 and 30 years.

Class 4. Three males and four females died from cephalitis; eleven males and twelve females from apoplexy; fifteen males and eleven females from paralysis; two males and one female from delirium tremens; four males from epilepsy; one male from tetanus; three males and three females from insanity; sixteen males and ten females from convulsions; and one male from injured spine. The deaths from convulsions occurred in subjects under 5 years of age; the case of tetanus between 10 and 15 years; those from apoplexy and paralysis principally between the ages of 60 and 80 years.

Class 5. One male died from pericarditis; one male and one female from aneurism; eleven males and twenty females from diseased heart; and one female from a rupture of a vessel near the heart. These deaths occurred principally in persons at ages between 55 and 75 years.

Class 6. Five males died from laryngitis; twenty-six males and twenty-eight females from bronchitis; two males and one female from pleuritis; thirty-one males and fifteen females from pneumonia; one male from asthma; and seven males and nine females from diseased lungs. The deaths from these diseases occurred principally in the months of January, February, March, and April of each year. The deaths from pneumonia principally in children; those from bronchitis at ages above 55 years.

Class 7. Five males and two females died from teething; one male from malignant tumour of the mouth; two females from stricture of the œsophagus; one male from gastritis; four males and two females from enteritis; one male and five females from peritonitis; three females from ascites; one female from hæmorrhage from the bowels; one male from tape-worm and debility; one male from diseased rectum; one female from tumour of the bowels; one female from malignant tumour of the abdomen; one male from fistula in ano; two males and two females from hernia; one male from ileus; two males and six females from diseased stomach; two males and three females from jaundice; four males and twelve females from diseased liver. With the exception of those from teething, these deaths occurred at nearly all ages, but the greater number were between the ages of from 50 to 80 years.

Class 8. Nephritis was fatal to one female; albuminuria to six males and three females; ischuria, to one male; diabetes, to two males; cystitis, to one male; diseased kidneys, to four males and three females.

Class 9. Two females died in childbirth; one female from disease of the organ of generation; the former of these were respectively between the ages from 20 to 25 years, and 40 to 45 years; the latter between 35 and 40 years.

Class 10. One male died from rheumatism; two males and one female from diseased bones.

Class 11. One female died from cyanosis; one from malformation from birth.

Class 14. Those reported to have died from age were more than 70 years old: three males and nine females were between 90 and 95 years; one male and three females were between 95 and 100 years.

Class 15. One male and one female are reported to have died from intemperance, between the ages of 25 and 45 years; one female, between 75 and 80 years, from excessive cold; one male, under five years, from poison; four males and four females from burns and scalds, five of these were under five years of age; three adult males from suicide by hanging; one female infant from suffocation; four males and one female from drowning: six males from fractures; one male, between 75 and 80 years, shot himself; and one male and one female, adults, are registered as having been feloniously killed.

The deaths, from all causes, have occurred at the following ages, viz.:—

| | Under 5 yrs. | 5 to 10 yrs. | 10 to 15 yrs. | 15 to 20 yrs. | 20 to 25 yrs. | 25 to 30 yrs. | 30 to 35 yrs. | 35 to 40 yrs. | 40 to 45 yrs. | 45 to 50 yrs. | 50 to 55 yrs. | 55 to 60 yrs. | 60 to 65 yrs. | 65 to 70 yrs. | 70 to 75 yrs. | 75 to 80 yrs. | 80 to 85 yrs. | 85 to 90 yrs. | 90 to 95 yrs. | 95 to 100 yrs. | Doubtful. | Totals. |
|---|---|---|---|---|---|---|---|---|---|---|---|---|---|---|---|---|---|---|---|---|---|---|
| Males .. | 145 | 22 | 8 | 17 | 25 | 29 | 14 | 17 | 17 | 18 | 23 | 15 | 25 | 30 | 35 | 28 | 19 | 14 | 3 | 1 | 1 | 506 |
| Females . | 109 | 28 | 5 | 21 | 19 | 14 | 9 | 18 | 9 | 22 | 13 | 17 | 24 | 36 | 26 | 42 | 18 | 16 | 9 | 3 | | 458 |
| Totals.. | 254 | 50 | 13 | 38 | 44 | 43 | 23 | 35 | 26 | 40 | 36 | 32 | 49 | 66 | 61 | 70 | 37 | 30 | 12 | 4 | 1 | 964 |

The mortality, and the nature of the diseases producing death are, to a certain extent, the most perfect index that can be procured of the health of a city or district. And although the proportion of deaths in the city of Canterbury has been above the lowest estimate, it is yet below the average of towns and densely populated districts; and as the tables for the two years, now recorded, prove the fatality has been in a large proportion among the aged population, and in but a comparatively small proportion among persons under twenty years of age; it does not appear from the tables of mortality, that the inhabitants of this city are afflicted with any disease peculiar to the locality; and, indeed, although a large proportion of the sickness, during the last twenty years, has fallen under my observation, I have not been able to discover, nor have I heard of other practitioners discovering, one of a peculiar endemic character; the one disease more endemic than others is, doubtless, that which affects the muscular or fibrous tissues, viz., rheumatism, which, however rarely, terminates fatally, unless by metastasis, or the induction of disease in some more vital organ, the heart being that, although comparatively rarely, most frequently so affected; and the records prove that a somewhat larger proportion of the deaths in Canterbury result from this disease, than in the country generally.

In conclusion, I have to acknowledge, in the preparation of this report, my obligations to the different local registrars through whose kindness, and with the consent of the Registrar-General, I have been enabled to examine, monthly, the records of births and deaths occurring in the city.

---

